# Density functional theory analysis for H_2_S adsorption on pyridinic N- and oxidized N-doped graphenes

**DOI:** 10.1039/d2ra00898j

**Published:** 2022-07-08

**Authors:** Takaya Fujisaki, Kei Ikeda, Aleksandar Tsekov Staykov, Hendrik Setiawan, Yusuke Shiratori

**Affiliations:** Institute of Multidisciplinary Research for Advanced Materials, Tohoku University 2-1-1 Katahira Aoba-ku Sendai Japan 980-8577 takaya.fujisaki.d5@tohoku.ac.jp; Institute for Materials Chemistry and Engineering and Integrated Research Consortium on Chemical Science (IRCCS), Kyushu University 744 Motooka, Nishi-ku Fukuoka Japan 819-0395; International Institute for Carbon-neutral Energy Research(WPI-I2CNER), Kyushu University 744 Motooka, Nishi-ku Fukuoka Japan 819-0395; Hydrogen Energy Systems, Graduate School of Engineering, Kyushu University 744 Motooka, Nishi-ku Fukuoka Japan 819-0395; Department of Mechanical Science and Engineering, School of Advanced Engineering, Kogakuin University 2665-1 Nakano-machi Hachioji Tokyo Japan 192-0015

## Abstract

Biomass discharged from primary industries can be converted into methane by fermentation. This methane is used for generating electricity with solid oxide fuel cells (SOFCs). This methane fermentation provides H_2_S, which reduces the efficiency of SOFCs even at a level as low as a few parts per million. It has been experimentally reported that a nitrogen (N)-doped graphene-based material known as pyridinic N removes H_2_S *via* an oxidation reaction compared with another graphene-based material known as oxidized N. To understand this experimental result, we investigated H_2_S adsorption on pyridinic N and oxidized N by a density functional theory analysis and further examined the activation barrier of dissociation reactions. We found that the adsorption of H_2_S on pyridinic N is more stable than that on oxidized N. In addition, the H_2_S dissociation reaction occurs only on pyridinic N.

## Abstract 2

We focus on graphene-based structures as promising adsorbents for H2S. DFT showed that the nitrogen-terminated structure (pyridinic N) showed better adsorption than the oxygen-terminated structure (oxidized N).

## Graphical abstract

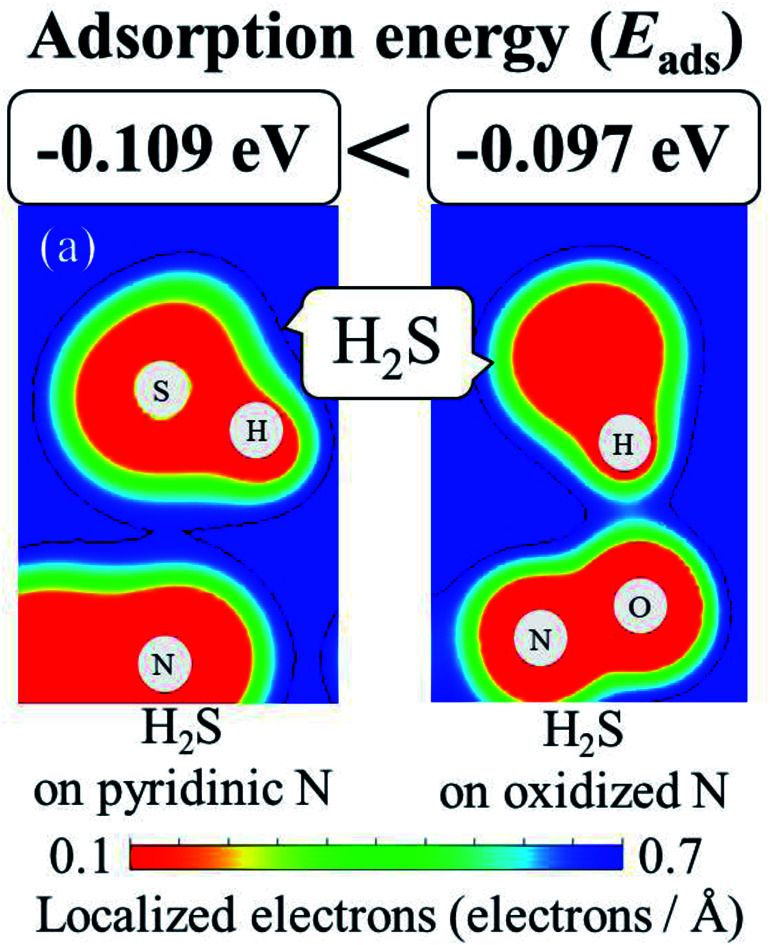

## Introduction

Approximately 80% of the world's ever-increasing energy demand is met by fossil fuels.^1,2^ They supply a significant amount of electrical energy to our society. These reserves are finite and many countries are actively promoting the use of renewable energy sources in anticipation of their depletion in the future.^3,4^ As constant generation of electricity from renewable energy sources is difficult, storage of the electrical energy using hydrogen as an energy carrier has received attention. Compared to conventional batteries, hydrogen is superior in storing energy on a large scale and for long time.^5^ To extract electricity from hydrogen, we can use fuel cells that emit only water and then have a low impact on the environment.^6^ The economic activity of producing, storing, and finally using hydrogen is called the hydrogen-based economy, and it is increasingly expected to be the economy that supplies electric energy to society in a sustainable way.^7–9^

One of the most environmentally friendly ways to produce hydrogen is water electrolysis using renewable energy. Some Organisation for Economic Cooperation and Development (OECD) countries such as Japan and Germany started demonstration.^10,11^ However, due to profitability issues, only the countries mentioned above attempt to make it a self-sustaining economic activity, while non-OECD countries do not.^12,13^ The non-OECD countries account for more than 80% of the total number of world countries,^14^ and the growth of energy demand in the future is expected to be much larger than that of OECD countries. Then, introducing the hydrogen-based economy with environmentally friendly ways to non-OECD countries is a major challenge to build a sustainable society on a global scale.^14^

**Fig. 1:**
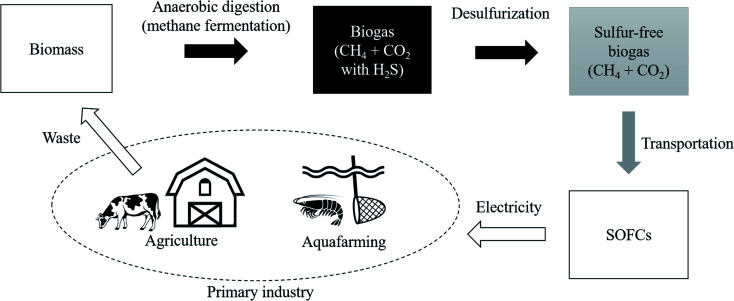
Material and energy circulation model using biomass.

As one of the ways to lead the challenge to success, combining biogas and fuel cells has attract ed attention.^15^ The flow diagram shown in Fig. 1 illustrates the use of biomass from primary industries such as agriculture and aquafarming. This biomass is converted into biogas by anaerobic digestion also known as methane (CH_4_) fermentation. This CH_4_ in biogas is used as an energy carrier of hydrogen and it is converted into electrical energy with solid oxide fuel cells (SOFCs). Following a reforming reaction shown in eqn (1) and (2), the H_2_ gas is extracted from CH_4_, with H_2_O, CO_2_, and CO.^16^ Then, hydrogen reacts with oxide ions (O^2−^) coming from the cathode side in fuel cells, as shown in eqn (3).^17^ This O^2−^ is supplied from O_2_ gas in the cathode side.

1 CH_4_ + H_2_O ⇆ CO + 3H_2_

2 CH_4_ + CO_2_ ⇆ 2CO + 2H_2_

3 H_2_ + O^2−^ ⇆ H_2_O

Regarding CO shown in eqn (1) and (2), it reacts with oxygen in the air and it is released into the atmosphere as CO_2_. One of the attractions of biomass is that it absorbs a certain amount of CO_2_ already at the plant growth stage. Therefore, the environmental impact of biomass is considerably smaller than that of CO_2_ generated when simply burning geo-derived resources such as coal and oil. This can be said to be the same advantage of bioethanol.^18,19^ In fact, the demonstration experiment has been started by Shiratori *et al.*, and they fermented biomass derived from aquafarming of shrimp in Vietnam.^20^ This initiative is a remarkable attempt to use CH_4_ as an energy carrier of hydrogen and introduce the hydrogen-based economy because non-OECD countries are mainly dominated by primary industries and they do not have a well-equipped hydrogen infrastructure.^21^

For the realization of the hydrogen-based economy using biogas, it is essential to increase the power generation efficiency of SOFCs. Ideally, it is the best way using pure CH_4_. However, actual biogas provided from biomass contains some amount of hydrogen sulfide (H_2_S),^22–24^ and it contaminates biogas in the process of anaerobic digestion process to form CH_4_.^25^ The amount of H_2_S is approximately 1 ppm and such amount reduces the efficiency of SOFCs^26^ and degrades even metallic materials such as pipes.^27,28^ Even if the amount of H_2_S is reduced to 0.001 ppm, the efficiency still decreases, and then H_2_S in biogas is recognized as a substance that should be removed as much as possible.^29^ For this purpose, some zeolites were reported to absorb it, but they have problems on profitability and durability.^30–34^ However, a more attractive method has been reported and it is called the Claus process.^35^ In this process, H_2_S is recovered externally as a single sulfur (S) *via* an oxidation reaction, as shown in eqn (4):^36–38^

4 2H_2_S + O_2_ ⇆ (2/*n*)S_*n*_ + 2H_2_O

To accelerate this process, metal oxides such as Fe_2_O_3_, V_2_O_5_, Al_2_O_3_, and TiO_2_ have been developed as catalysts.^39–42^ However, these metal oxides are corrosive to H_2_S and this causes lower efficacy of the oxidation process.^43^ Instead of these metal oxides, nanocarbons that are nano-scale carbon materials have recently attracted attention. Surprisingly, it can be useful in a wide temperature range from room temperature to even around 900 °C. Moreover, it has a high surface area with pore structures and unique surface function.^44–49^ Because nanocarbons can be utilized without metal-based active sites, it is expected that the reduction of catalytic performance due to H_2_S poisoning is avoided. The first reaction to remove H_2_S on nanocarbons is shown in eqn (5) as a dissociation reaction. Then, HS^−^ reacts with oxygen radicals (O*) and sulfur (S) is recovered following eqn (6):^50^

5 H_2_S ⇆ H^+^ + HS^−^

6 HS^−^ + O* ⇆ S + OH^−^

Because the carbon site itself in nanocarbons is inactive to remove H_2_S, substitution of heteroatoms with some carbon sites is a reasonable approach to make the inactive site active one. It was first shown by Chizari *et al.* that a nitrogen (N)-doped carbon nanotube had excellent activity for the oxidation reaction of H_2_S.^51^ Since then, several researchers have started studies on N-doped nanocarbons focusing on detailed microstructures,^52^ controlling the nitrogen content,^53^ investigating the effect of caustic alkali impregnating agents,^54^ and developing the preparation process for mesoporous materials.^55^ Furthermore, it is interesting to report that the edge site of N-doped graphene sheets promotes the oxidation reaction of H_2_S than the ordered plane (*cf.* pure graphene structures).^56,57^ Such sites might act as the origin of the high catalytic performance for H_2_S selective oxidation. Then, focusing on hetero atoms such as N-doped nanocarbons becomes one of the most important approaches. In other words, finding the best active site promoting the oxidation is the common target. However, because the guideline is still not clear, the industrial-scale application has been hampered.

**Fig. 2:**
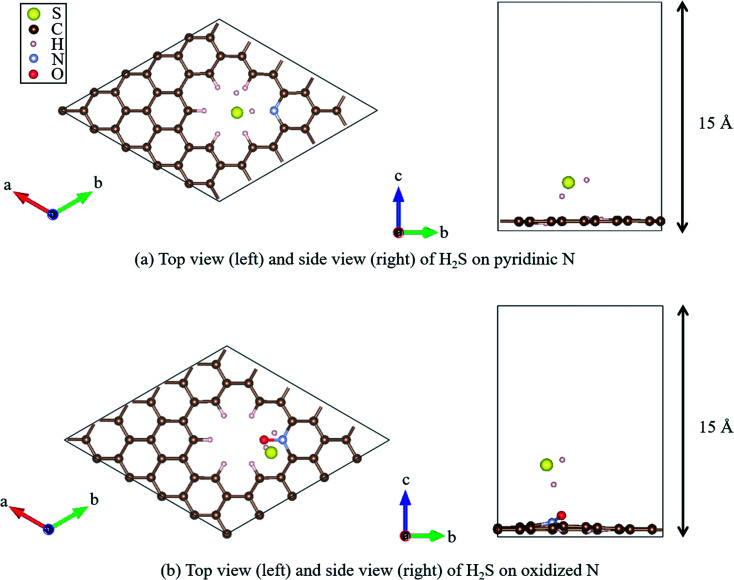
Top and side views of the geometry optimized structure of H_2_S on (a) pyridinic N and (b) oxidized N.

Recently, Shiyan *et al.* have conducted comparative experiments on the effects of N- and O-doped nanocarbons on the oxidation reaction of H_2_S. They conducted experiments demonstrating that pyridinic N (Fig. 2(a)), in which N is doped into nanocarbon and hydrogen is bonded to the nitrogen as a terminal atom, promotes the oxidation reaction the most. However, another graphene-based structure known as oxidized N (Fig. 2(b)), in which an oxygen atom is substituted for the hydrogen bonded next to the nitrogen atom in pyridinic N, showed the least enhancement of the oxidation reaction of H_2_S. To understand the difference in enhancement between pyridinic N and oxidized N at an atomic level, they attempted a density functional theory (DFT) approach. They reported that the adsorption of HS^−^ on pyridinic N was the highest compared with that on oxidized N that showed least adsorption.^58^ However, this previous study has the following demerit: HS^−^ is a dissociate of H_2_S, and the stability of HS^−^ cannot be discussed unless it is shown that H_2_S gets adsorbed onto pyridinic N and oxidized N. Therefore, the aim of this study is to clarify the adsorption properties of H_2_S on pyridinic N and oxidized N by DFT.

## Computational details

All DFT calculations were performed using the Vienna *Ab initio* Simulation Package (VASP).^59–61^ The Perdew–Burke–Ernzerhof (PBE)^62^ exchange-correlation functional was employed based on the projector augmented wave (PAW) method.^61^ The cutoff energy for the plane wave basis was set to 400 eV, referring to a previous study.^63^ First, a 4 × 4 graphene sheet was constructed and optimized using 5 × 5 × 1 Monkhorst–Pack *k*-point meshes. Moreover, the spin polarization was applied. For the projector augmented wave (PAW) method, pseudopotentials with valence states of C (2s^2^, 2p^2^), N (2s^2^, 2p^3^), S (3s^2^, 3p^4^), O (2s^2^, 2p^4^), and H (1s) were used. All ionic positions were optimized by a conjugate gradient method until the forces on each ion were below 10^−3^ eV Å^−1^. The electronic energy was converged to less than 10^−5^ eV. Then, after the geometrical optimization of the 4 × 4 graphene sheet under the above-mentioned conditions, the length of the C–C bond was found to be 1.426 Å, which is in good agreement with previous results.^64–66^ In addition, the internal angles of the six-membered ring of graphene structures were within the range of 120° to ± 0.025%, which is also in good agreement with the results of previous studies.^67,68^ Next, with the optimized graphene sheet spreading to *a*- and *b*-axis directions, we added a vacuum slab on it by making 15 Å height in the *c*-axis direction. Then, geometry optimization was performed while keeping the vacuum slab. From the optimized graphene sheet with a vacuum slab, a six-membered ring structure consisting of six carbons was removed. Then, hydrogen atoms were introduced to terminate the remaining carbon atoms. The edge of the graphene sheet is reported to be bonded with hydrogen atoms,^69^ which prevents the carbons from becoming radicalized and destabilized. Finally, the carbon site was replaced with a nitrogen atom (N), as shown in Fig. 2(a), and with nitrogen and oxygen, as shown in Fig. 2(b). The respective structure is called pyridinic N and oxidized N, respectively.^58^ They were visualized using VESTA.^70^

Fig. 2(a) and (b) show that hydrogen sulfide (H_2_S) was adsorbed onto pyridinic N and oxidized N, and H_2_S itself was optimized. For this, H_2_S was first structurally optimized in vacuum. H_2_S has a bent structure with lone-pair electrons in the S atom, and the calculated S–H bond length is 1.35 Å (1.35 Å and 1.33 Å). Moreover, the H–S–H angle was found to be 91.2°(91.6°and 92.2°), which is in good agreement with the previous experiment and calculated values in parentheses.^71^ This single molecule of H_2_S in vacuum was structurally optimized with 1 × 1 × 1 Monkhorst–Pack *k*-point meshes. For each of the above-mentioned pyridinic N and oxidized N, H_2_S was placed on a nitrogen atom and the structure was optimized. The position of H_2_S was referred from the previous study by Shiyan *et al.*^72^

To accurately determine the stability of H_2_S on pyridinic N and oxidized N, we considered adsorption energy (*E*_ads_).^73,74^ It is defined in eqn (7), which is the sum of the interaction energy (*E*_int_) in eqn (8), and deformation energy (*E*_def_) shown in eqn (9). The deformation energy is obtained as follows: H_2_S and pyridinic N form the most stable structure when they are placed in vacuum with no other atoms around them. However, H_2_S and pyridinic N deforms when they are close to each other due to their interaction. The change in the electrostatic potential at this time is defined as the deformation energy. The interaction energy (*E*_int_) and deformation energy (*E*_def_) shown in eqn (8) and (9) are the case for pyridinic N with H_2_S. When the adsorption energy was calculated as negative, H_2_S gets adsorbed onto pyridinic N stably.

7 *E*_ads_ = *E*_def_ + *E*_int_

8 *E*_int_ = *E*_H_2_S+pyrindinic N_ − (*E*_pyridinic N_ + *E*_H_2_S_)

9 *E*_def_ = *E*_def_H_2_S_ + *E*_def_pyridinic N_

*E*_H_2_S+pyrindinic N_, *E*_pyridinic N_, and *E*_H_2_S_ are the electrostatic potential for pyridinic N and adsorbed H_2_S, pyridinic N, and single H_2_S, respectively.

Moreover, the minimum energy paths and the energy barriers for H_2_S dissociation on graphene-based structures were obtained by the climb image nudged elastic band (NEB) method. Five intermediate images were used to sample the reaction path between the initial adsorption state and the final dissociated state. The spring constant of the NEB calculations was 5.0 eV Å^−2^ and the NEB force convergence criteria was 0.01 eV Å^−1^ using 3 × 3 × 1 Monkhorst–Pack *k*-point meshes.

## Results and discussion

The calculated adsorption energies for H_2_S on pyridinic N and oxidized N are given in Table 1. The electrostatic potentials are summarized in Table 2. As shown in Table 1, H_2_S on pyridinic N shows −0.117 eV as the adsorption energy, and this number is 21% lower than that of oxidized N as −0.097 eV. This indicates that H_2_S is more stably adsorbed onto pyridinic N than onto oxidized N, indicating that pyridinic N is a more favorable material than oxidized N to proceed the oxidation reaction (2H_2_S + O_2_ ⇆ (2/*n*) S_*n*_ + 2H_2_O), as shown in eqn (4). Shiyan *et al.* experimentally reported the same trend,^72^ and the result of our study supports their result successfully by DFT. Notably, oxidized N does not adsorb H_2_S as readily as pyridinic N, but this indicates that H_2_S on oxidized N can be desorbed relatively easily by external energies such as heat. This suggests that the structure of oxidized N could be applied to the reusable desorption sheet to remove H_2_S.

**Table 1:** Adsorption, interaction, and deformation energies of H_2_S on pyridinic N and oxidized N

	On pyridinic N (eV)	On oxidized N (eV)
Adsorption energy	−0.117	−0.097
Interaction energy	−0.129	−0.114
Deformation energy of H_2_S	0.003	0.002
Deformation energy of pyridinic N/oxidized N	0.009	0.015

**Table 2:** Adsorption, interaction, and deformation energies of H_2_S on pyridinic N and oxidized N^a^

Structure	Energy (eV)
Pyridinic N	−420.563
Oxidized N	−425.468
H_2_S with pyridinic N	−431.879
H_2_S with oxidized N	−436.778
Pyridinic N non-relaxed	−420.554
Oxidized N non-relaxed	−425.453
H_2_S (∠HSH = 92.06°)	−11.192
H_2_S (∠HSH = 91.886°)	−11.193
H_2_S (∠HSH = 91.20°)	−11.195

a The values of “pyridinic N non-relaxed” and “oxidized N non-relaxed” mean the electrical potential of pyridinic N and oxidized N without performing geometry optimization by removing H_2_S from “H_2_S with pyridinic N” and “H_2_S with oxidized N”, respectively. In addition, the energy for each angle of H_2_S is listed in the last three lines.

**Fig. 3:**
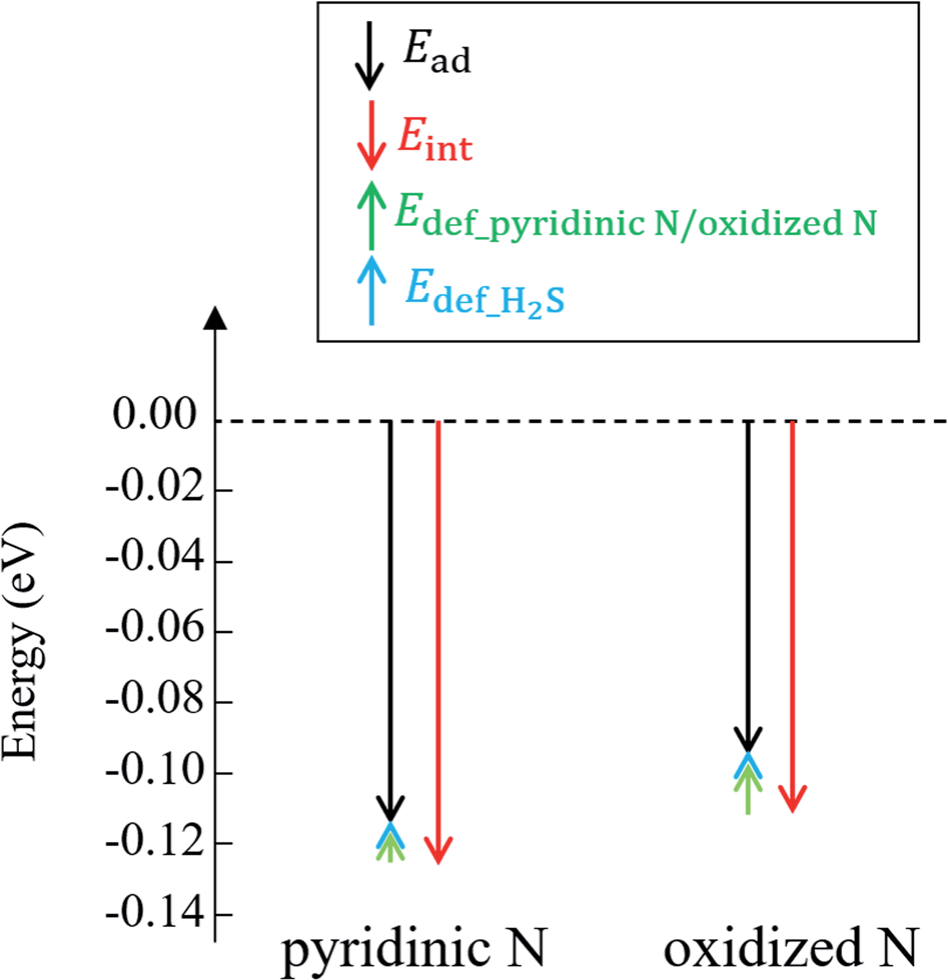
Adsorption energy (*E*_ads_) and interaction energy (*E*_int_) between H_2_S and pyridinic N/oxidized N. The deformation energies of pyridinic N (*E*_def_pyridinic N_), oxidized N (*E*_def_oxidized N_), and H_2_S are also shown.

The reason why H_2_S adsorption occurs more favorably on pyridinic N than on oxidized N is explained as follows. As mentioned before, the adsorption energy is expressed as the sum of the interaction energy, shown in eqn (8), and the deformation energy, also shown in eqn (9). Their values are presented in Table 1 and their amount is graphically visualized in Fig. 3.

**Fig. 4:**
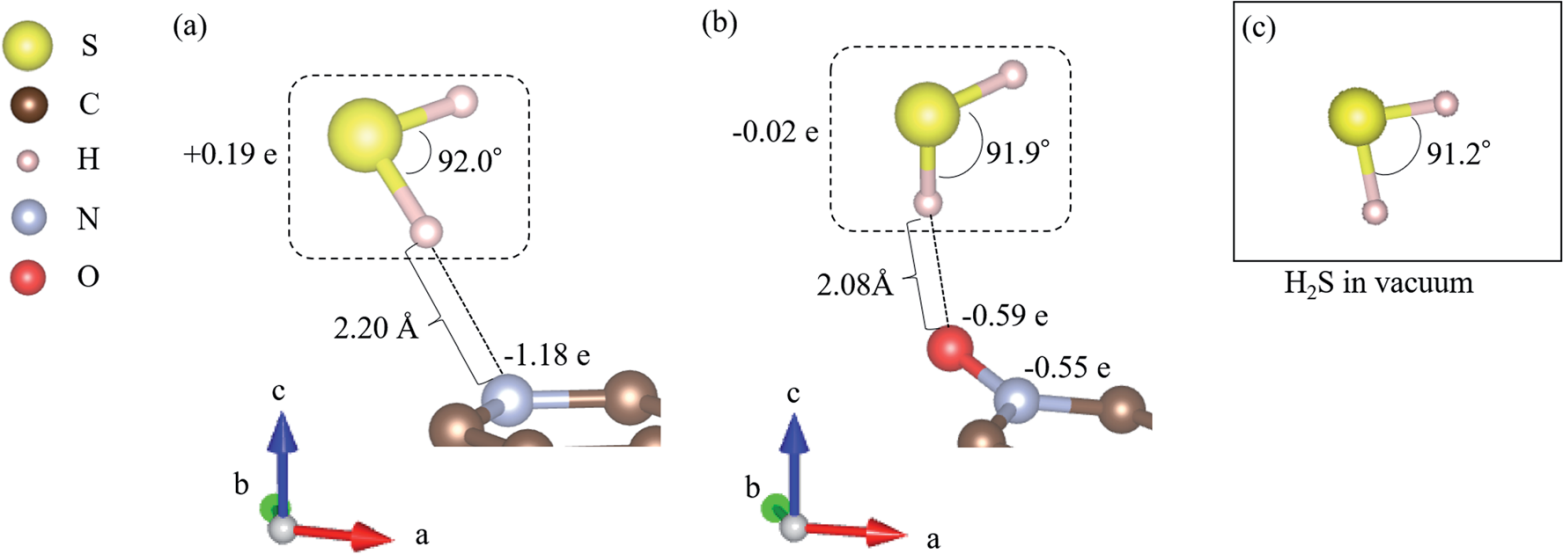
Adsorption energy (*E*_ads_) and interaction energy (*E*_int_) between H_2_S and pyridinic N/oxidized N. The deformation energies of pyridinic N (*E*_def_pyridinic N_), oxidized N (*E*_def_oxidized N_), and H_2_S are also shown.

As shown in Fig. 3, it is clear that the effect of the interaction energy is much larger than that of the deformation energy in determining the adsorption energy. The first factor that determines the amount of the interaction energy is reported with the idea of attractive force (intermolecular force) that acts between materials caused by the transfer of charge.^75,76^Fig. 4 shows electric charges of adsorbed H_2_S and its surrounding atoms: N and O. Every charge was obtained by Bader charge analysis.^77^ This figure shows pyridinic N (Fig. 4(a)) and oxidized N (Fig. 4(b)) with adsorbed H_2_S, respectively, and each structure has already been geometry optimized. Fig. 4(c) shows H_2_S placed in vacuum. From pyridinic N and oxidized N, H_2_S is adsorbed approximately 2 Å away, and this value has good agreement compared to previous studies reporting the adsorption distances between molecules and graphene-based structures.^78,79^ As shown in Fig. 4(a), the charge of the entire H_2_S was calculated as +0.19 e (electron), and the charge of the N atom of pyridinic N adjacent to H_2_S was polarized to −1.18 e. However, as shown in Fig. 4(b), H_2_S is polarized to −0.02 e, and the charges of O and N of oxidized N are polarized to −0.59 e and −0.55 e. The difference in charge polarization can be explained by the difference in electronegativity whose order is O > N > S > C > H in the atoms, as shown in Fig. 4.^80^ Because the N atom in pyridinic N has the highest electronegativity, N has the largest polarization as −1.18 e. Such large polarization is the result of attracting electrons, and this makes the charge of H_2_S as +0.19 e. This suggests that the intermolecular force between pyridinic N and H_2_S contributes to the adsorption energy of H_2_S to make the H_2_S stable. However, the charge on the O atom of oxidized N is negatively polarized as −0.59 e, and that of H_2_S is also negatively polarized as −0.02 e. This indicates that repulsive forces are at work between them. Despite the presence of repulsive force between H_2_S and oxidized N, the reason why the adsorption energy is negative as shown in Table 1 can be the formation of ionic or covalent bonds that have a stronger binding force than the repulsive force generated by intermolecular force. Therefore, in addition to pyridinic N and H_2_S, the covalent nature between oxidized N and H_2_S was investigated as follows.

**Fig. 5:**
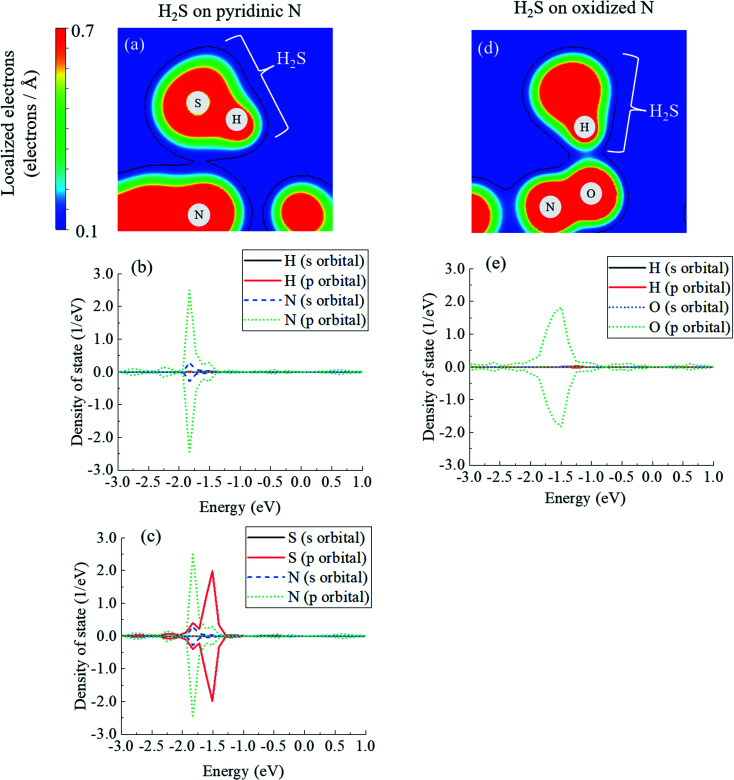
(a) Electron mapping for H_2_S on pyridinic N, including density of states of (b) H–N and (c) S–N electrons. (d) Electron mapping of H_2_S on oxidized N, and (e) density of states of H–O.

In fact, the relationship between the covalent nature and the interaction energy has been discussed in several papers. Wang *et al.* reported the bonding nature of PbO molecules on the aluminium surface, and they showed that the magnitude of covalent bonding contributes to the value of interaction energy.^81^ Moreover, Zhou *et al.* mentioned that ammonia molecules adsorbed onto SiSe layers showed more stability with greater covalency.^82^ In addition, covalent bonding can be explained by the amount of electrons between atoms, and the bonding between zirconium and oxygen atoms seems to be greater than that between cerium and oxygen atoms in perovskite structures.^83^ Considering these previous studies, the magnitude of the covalency in terms of the number of electrons between atoms has a significant effect on the interaction energies. Fig. 5(a) shows the electron mapping obtained by slicing the coordinates of the following three atoms: the N atom of pyridinic N, the S atom of H_2_S, and the H atom of H_2_S. Fig. 5(b) describes the density of states of the H atom in H_2_S and the N atom in pyridinic N, which are neighbouring atoms, and Fig. 5(c) shows the density of states of S in H_2_S and N in pyridinic N. In Fig. 5(a), it seems that there is little sharing to create a covalent bonding between the H atom and the N atom. However, as shown in Fig. 5(c), the s- and p-orbitals of the N atom in pyridinic N clearly overlap on the p-orbital band structure of the S atom. From the above, it is shown that the electrons between H_2_S and pyridinic N shown in Fig. 5(a) make a covalent bond between S of H_2_S and the N of pyridinic N. This electron sharing stabilizes H_2_S on pyridinic N and it helps determining the number of interaction energy to be −0.129 eV, as shown in Table 1. However, Fig. 5(d) shows the electron mapping of H_2_S adsorbed onto oxidized N using the coordinates of the O and N atoms of oxidized N, and the H atom of H_2_S. From this figure, it is observed that there are electrons between the O atom of oxidized N and the H atom of H_2_S. This number of electrons in Fig. 5(d) seems to be larger than that of electrons between pyridinic N and H_2_S, as shown in Fig. 5(a). However, Fig. 5(e) shows that s- and p-orbitals of the H atom in H_2_S hardly overlap with the s- and p-orbitals of the O atoms in oxidized N. The is the proof that the type of bond between H_2_S and oxidized N is ionic bond. Focusing on the p-orbital of the O atom, the bond between O and H appears to be formed as a covalent bond, as shown in Fig. 5(d), as the density of state in the p-orbital of O is distributed over a wide range of energy, as shown in Fig. 5(e) and then electrons of the p-orbital are widely delocalized around the O atom. In other words, there is no covalent but mainly ionic bond between H_2_S and oxidized N. Although there was repulsive intermolecular force between H_2_S and oxidized N, this ionic bonding between them makes H_2_S stable on oxidized N, which results in the negative adsorption energy, as shown in Table 1. As described above, the difference in the interaction energies presented in Table 1 can be attributed to the difference in the degree of covalent bonding property between H_2_S and pyridinic N or oxidized N.

**Fig. 6:**
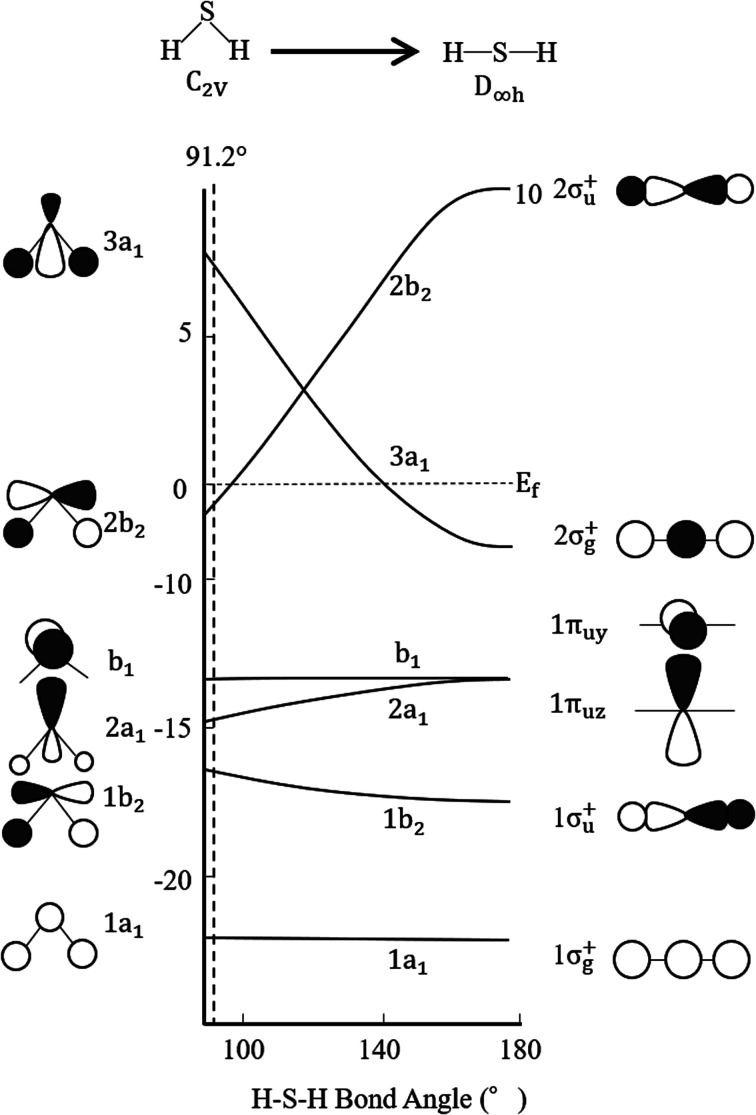
Walsh diagram of H_2_S. Black and white lobes show positive and negative wave functions, respectively.

Finally, regarding the deformation energy, it is significantly smaller relative to the interaction energy, as shown in Table 1 and Fig. 3. However, to exactly determine the adsorption energy in this study, it is important to explain the effect of such minor energy contribution. First, the deformation energies of pyridinic N and oxidized N after adsorption of H_2_S were calculated as +0.009 eV and +0.015 eV, respectively. The reason for the higher deformation energy of oxidized N is that the O atom is attracted to the H atom of H_2_S by intermolecular force, as shown in Fig. 1 and 4. As for the deformation energy of H_2_S, the value for H_2_S on pyridinic N is +0.003 eV, and that for oxidized N is +0.002 eV. The reason for the difference is that the bond angle of H_2_S on pyridinic N is 92.0°and this value is larger than the bond angle (91.9°) on oxidized N, as shown in Fig. 4(a) and (b). In fact, both angles are larger than the angle of H_2_S in vacuum (91.2°), as shown in Fig. 4(c). The origin of such angle change has already been well discussed in previous studies and the cause comes from the result of charge transfer in molecules. Bajdich *et al.* reported that charge transfer occurs to CO_2_ adsorbed on the Au surface, leading to a change in bond angle.^84^ Indeed, as shown in Fig. 4(a) and (b) in our study, the charge transfer between H_2_S and pyridinic N is larger than that between H_2_S and oxidized N. Therefore, the bond angle of pyridinic N is considered to be larger than that of oxidized N. The bond angle change is also explicable from the viewpoint of the electronic structure of Walsh diagram, as shown in Fig. 6.^85^ The diagram shows that the energy of orbitals b_1_ and 2a_1_ increases as the angle of H_2_S increases in the narrow range of 91.2 to 92.0°. The above-mentioned difference can be clarified if we can describe the density of state with sufficiently high accuracy. However, the VASP used in this study was unable to follow the change in the Walish diagram within 1.0° of the H_2_S bond angle. Therefore, it is significantly desirable to have a method that can discuss the change in the density of states for a very narrow range of bond angle, which is less than 1°.

**Fig. 7:**
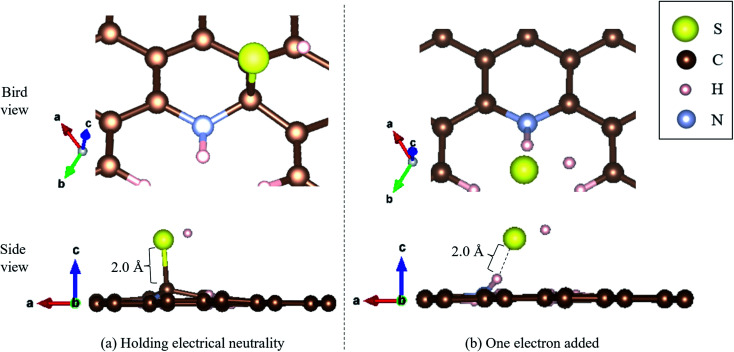
H_2_S dissociated as HS^−^ and H^+^ on pyridinic N under (a) holding electrical neutrality, and (b) one electron added condition.

**Fig. 8:**
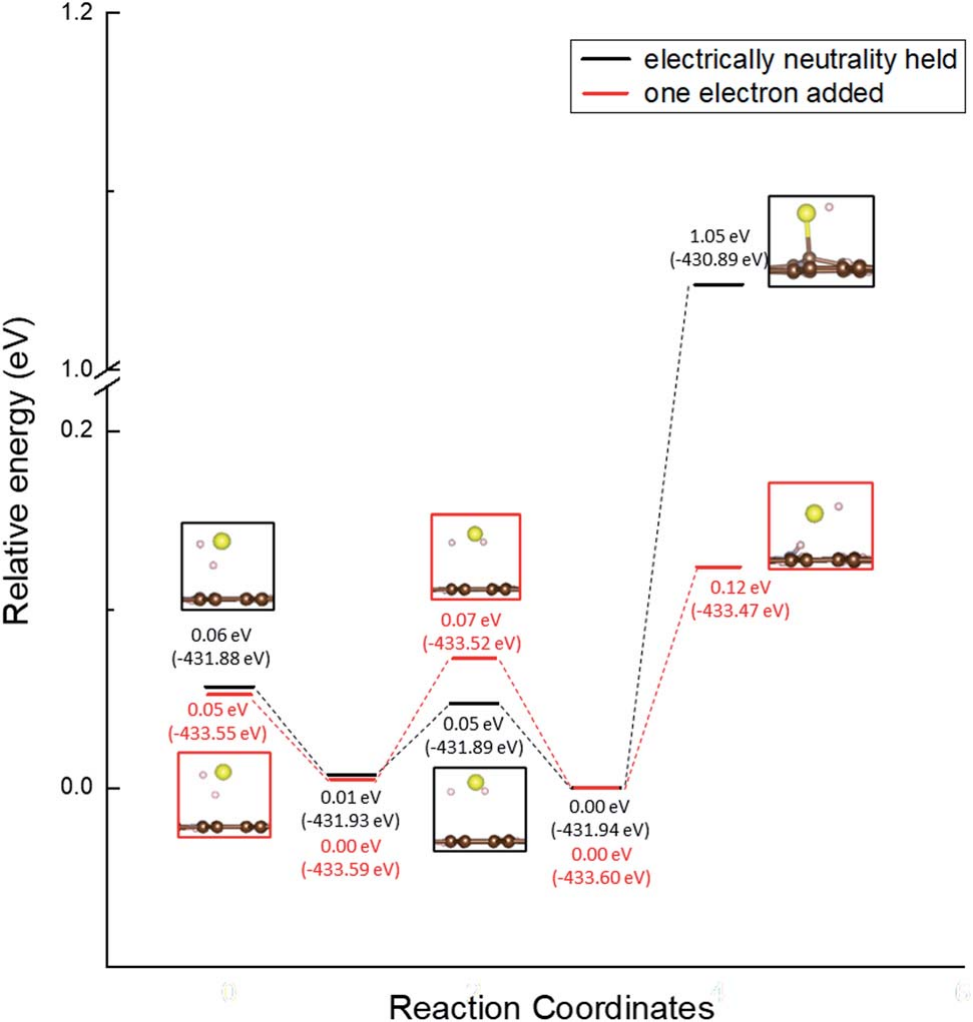
Activation barrier of the H_2_S dissociation reaction on pyridinic N under the conditions of holding electrical neutrality and adding one electron.

Moreover, we determined the activation barrier for H_2_S molecules to dissociate on the graphene structure. Fig. 7 shows H_2_S dissociated as HS^−^ and H^+^ on Pyridinic N. Fig. 7(a) shows the case where H_2_S is dissociated while maintaining the electrically neutral condition, and Fig. 7(b) shows the case where one electron is added to the entire system. Both cases show cases where the most dissociated H_2_S is stabilized at several locations around the nitrogen atom. The electron-added system shown in Fig. 7(b) was chosen for investigation because of the reduction reaction. A previous study has also considered the possibility that pyridinic N was reduced by experimental residual reducing gases such as carbon monoxide and hydrogen gas.^72^ In fact, a previous study also suggested there is some amount of oxygen in experimentally prepared pyridinic N.^72^ With the initial structure shown in Fig. 2(a) and final structures shown in Fig. 7 of pyridinic N, the activation barrier was calculated as shown in Fig. 8. Focusing on the final structure and when the electroneutrality is maintained, the final structure is about 1.05 eV larger than that of the previous structure. However, when a single electron was added into the entire system, the final structure shows 0.12 eV larger activation barrier than the previous structure. This value (0.12 eV) is about one-tenth the size of the structure under electroneutrality. This indicates that the hydrogen sulfide dissociation was significantly enhanced by the reducing gas.

However, H_2_S is always stable as a molecule on oxidized N, and the dissociation reaction could not be reproduced. Although a previous study had raised the possibility that a water film could cause H_2_S to dissociate,^86^ the separation of H_2_S into HS^−^ and H^+^ by water molecules could not be reproduced by our investigation. Chemical reactions such as the above-described dissociation reaction do not occur because the interaction energy between H_2_S and oxidized N is −0.114 eV, which is so small that there might be contribution of hydrogen bonding as well as ionic bonding. However, as shown in Fig. 5(e), there is a slight overlap of electrons between the O p-orbital and the H p-orbital, making it difficult to precisely separate the effect of hydrogen bonding from that of ionic bonding. As mentioned above, we could not find any tendency for H_2_S to react with oxidized N in a divergent manner. Therefore, we conclude that oxidized N does not contribute to the dissociation reaction for H_2_S, but rather has the effect of preventing the dissociation reaction.

## Conclusions

In this study, the adsorption energies of H_2_S on pyridinic N and oxidized N have been precisely investigated by examining the interaction and deformation energies by DFT. Our result indicated that H_2_S gets adsorbed more preferably onto pyridinic N than onto oxidized N. This indicates that pyridinic N is a significant material for the oxidation reaction of H_2_S. The above-mentioned difference is explicable because the interaction energy of H_2_S with pyridinic N is lower than that with oxidized N, which can be inferred from the difference in intermolecular forces and covalency properties. As for the deformation energy, it is remarkably small compared to the interaction energy, but oxidized N has a higher deformation energy than pyridinic N, which is due to the intermolecular force between the O atom in oxidized N and the H atom in H_2_S. It was also shown that the bond angle of H_2_S is larger on pyridinic N than on oxidized N due to charge transfer after adsorption of H_2_S.

Furthermore, the activation barrier for the dissociation of H_2_S (H_2_S ⇆ H^+^ + HS^−^) on pyridinic N and oxidized N was investigated and found to be 1.05 eV on pyridinic N. Moreover, when one electron was added considering the reducing atmosphere, the activation barrier on pyridinic N was found to be 0.12 eV, which became very smaller. However, we could not reproduce the reaction of H_2_S dissociation on oxidized N, and concluded that only pyridinic N contributes to the H_2_S dissociation reaction.

## Author contributions

T. F. and Y. S. designed this project. K. I. and A. T. S. provided valuable advice for proceeding this research. Also, H. S. gave useful viewpoint from experimental aspect. The manuscript was written through contributions of all authors. All authors have given approval to the final version of the manuscript.

## Conflicts of interest

There are no conflicts to declare.

## Supplementary Material
